# Editorial for Special Issue “Dynamics and Mechanics in Two-Dimensional Nanostructures: Simulation and Computation”

**DOI:** 10.3390/nano13030400

**Published:** 2023-01-18

**Authors:** Kai Ren, Bin Ding, Gang Zhang

**Affiliations:** 1 School of Mechanical and Electronic Engineering, Nanjing Forestry University, Nanjing 210037, China; 2 Institute of Solid Mechanics, School of Aeronautic Science and Engineering, Beihang University, Beijing 100191, China; 3 Institute of High Performance Computing, Agency for Science, Technology and Research (A*STAR), Singapore 138632, Singapore

Two-dimensional (2D) materials have completely different thermal transport characteristics from bulk materials. This is mainly induced by their phonon properties [1]. Phonon performance might be considered the intrinsic dynamic mechanism of 2D materials. In contrast to acoustic phonons, optical phonon modes can be used to evaluate interlayer coupling, spin–orbit coupling, magneto-optic coupling, and the number of atomic layers through Raman spectroscopy measurement. Thus, the dynamics of 2D materials are critical for electronic [2], magnetic [3,4,5], and thermal [6] performance.

In this Special Issue, Liu et al. [7] used ab initio molecular dynamics (AIMD) simulations to investigate the thermal properties of the Janus monolayers SnXY (X, Y = O, S, Se). A system with higher thermal stability exhibits a smaller difference in the bond length of Sn–X and Sn–Y, which is consistent with the orders obtained after comparing their electron localization functions and atomic displacement parameters. A simple rule to quickly predict the maximum temperature up to which the Janus monolayer can stably exist, where the only input was ADP calculated using second-order interatomic force constants rather than time-consuming AIMD simulations at various temperatures, was proposed.

Other investigations in this Special Issues also demonstrate effective methods of calculation. Altbir et al. presented an analysis of skyrmion dynamics, considering Dzyaloshinskii–Moriya interactions in an STNO device with double-disk geometry [8], using numerical simulations. Additionally, three regimes were addressed as a function of the geometric parameters and electric current density: (1) the skyrmion is annihilated at the system’s border; (2) the skyrmion moves in a non-circular trajectory, alternating its position between the two disks; and (3) the skyrmion only rotates inside a one-disk subsystem. Furthermore, the dynamics of two skyrmions nucleated in a double-disk structure were explored, which explained the different forces that skyrmions are subject to. These are shown in a state diagram of the dynamical states that allow an adequate understanding of the associate phenomena. 

Double quantum dots were constructed using an MoS_2_-based heterostructure possessing a 1T-phase embedded in a 2Hphase with the aim of investigating the feasibility of controlled-NOT gate operation with Coulomb interactions. The Hamiltonian of the system was addressed. Then, the dynamics of states were investigated using the Crank–Nicolson method in the potential model and the fourth order Runge–Kutta method in the matrix model. This showed that the constructed matrix model could be used to simulate the dynamical behaviors of two interacting double quantum dots with lower computational resources [9]. In another work, by Wang et al. [10], the mechanical properties of pure graphene nanoribbons and graphene nanoribbons with vacancy defects were calculated using the molecular dynamics method. They found that the vibration frequency not only decreased significantly with the increase in nanoribbon length but also with the increase in vacancy concentration.

The surface-enhancement of the Raman signal was investigated by Lombardi and co-workers [11]. Vibronic coupling of the allowed molecular transitions, with charge-transfer transitions between the molecule and the substrate, are responsible for the surface-enhancement of the Raman signal in semiconductor substrates. Such expression of the Raman enhancement in monolayer graphene was proven to be dependent on the square of the derivative of the density of states of the graphene. This allows people to maximize the Raman intensity by carefully aligning the doping level of the graphene substrate with the charge-transfer transition.

In conclusion, we would like to thank the authors for providing their important contributions to this Special Issue. We greatly appreciate Olivia Sun for organizing this Special Issue, as well as the whole editorial team of *Nanomaterials*, for their great support and kind cooperation. We sincerely hope that the readers will enjoy reading this Special Issue.
